# Multiple Origins of Foot-and-Mouth Disease Virus Serotype Asia 1 Outbreaks, 2003–2007

**DOI:** 10.3201/eid1507.081621

**Published:** 2009-07

**Authors:** Jean-Francois Valarcher, Nick J. Knowles, Valery Zakharov, Alexey Scherbakov, Zhidong Zhang, You-Jun Shang, Zai-Xin Liu, Xiang-Tao Liu, Aniket Sanyal, Divakar Hemadri, Chakradhar Tosh, Thaha J. Rasool, Bramhadev Pattnaik, Kate R. Schumann, Tammy R. Beckham, Wilai Linchongsubongkoch, Nigel P. Ferris, Peter L. Roeder, David J. Paton

**Affiliations:** Institute for Animal Health, Pirbright, UK (J.-F. Valarcher, N.J. Knowles, Z. Zhang, N.P. Ferris, D.J. Paton); FGI All-Russian Research Institute for Animal Health, Vladimir, Russian Federation (V. Zakharov, A. Scherbakov); Lanzhou Veterinary Research Institute, Lanzhou, People’s Republic of China (Y.-J. Shang, Z.-X. Liu, X.-T. Liu); Project Directorate on Foot-and-Mouth Disease, Mukteswar-Kumaon, India (A. Sanyal, D. Hemadri, C. Tosh, T.J. Rasool, B. Pattnaik); Plum Island Animal Disease Center, Orient Point, New York, USA (K.R. Schumann, T.R. Beckham); Regional Reference Laboratory for Foot and Mouth Disease in South East Asia, Pakchong, Thailand (W. Linchongsubongkoch); Food and Agriculture Organization of the United Nations, Rome, Italy (P.L. Roeder); 1 Current affiliation: Swedish University of Agricultural Sciences, Uppsala, Sweden.; 2 Current affiliation: Indian Veterinary Research Institute, Bhopal, India.; 3 Current affiliation: Animal Production and Breeding, Krishi Bhavan, New Delhi, India.; 4 Current affiliation: Texas Veterinary Medical Diagnostic Laboratory, College Station, Texas, USA.; 5 Current affiliation: Taurus Animal Health, Hampshire, UK.

**Keywords:** Picornaviridae, Aphthovirus, foot-and-mouth disease virus, serotype Asia 1, surveillance, epidemiology, phylogeny, sequencing data, viruses, research

## Abstract

We investigated the molecular epidemiology of foot-and-mouth disease virus (FMDV) serotype Asia 1, which caused outbreaks of disease in Asia during 2003–2007. Since 2004, the region affected by outbreaks of this serotype has increased from disease-endemic countries in southern Asia (Afghanistan, India, Iran, Nepal, Pakistan) northward to encompass Kyrgyzstan, Tajikistan, Uzbekistan, several regions of the People’s Republic of China, Mongolia, Eastern Russia, and North Korea. Phylogenetic analysis of complete virus capsid protein 1 (VP1) gene sequences demonstrated that the FMDV isolates responsible for these outbreaks belonged to 6 groups within the Asia 1 serotype. Some contemporary strains were genetically closely related to isolates collected historically from the region as far back as 25 years ago. Our analyses also indicated that some viruses have spread large distances between countries in Asia within a short time.

## Abstract 2

Viruses in 6 genetic groups have caused recent outbreaks in Asia.

Foot-and-mouth disease virus (FMDV) is an *Aphthovirus* within the family *Picornaviridae* that infects domestic and free-ranging cloven-hoofed mammals. The virus occurs as 7 serotypes, and immunity after vaccination or after infection is type specific (*1*–*3*). Diversity is also apparent within serotypes, and phylogenetic studies have proved useful for tracing the origin of foot-and-mouth disease (FMD) outbreaks (*4*).

FMDV is highly contagious, and this, together with its ability to infect different hosts and to exist as multiple types and variants, makes FMD difficult to control and a severe constraint to international trade of livestock and their products. FMD is endemic to regions of South America and large areas of Africa and Asia, and it can readily cross international boundaries to cause epidemics in previously disease-free areas (*5*). High densities of ruminants and swine in Asia create potential reservoirs of virus maintenance and evolution not influenced by control measures. Intense trading of animals and their products from these reservoirs results in widespread dissemination of viruses within and outside this continent. Therefore, epidemiologic surveillance of FMD in Asia is essential for the timely detection of the emergence of new strains that could threaten neighboring countries (*6*) and for selecting the most appropriate vaccine strains for use and storage in emergency vaccine reserves (*7*).

Globally, FMDV serotypes O and the A are the most prevalent. However, Asia has its own unique serotype, Asia 1, first detected in samples collected in India in 1951 through 1952 (*8*) and Pakistan in 1954 (*9*). The primary serotype-endemic region for Asia 1 seems to be the Indian subcontinent (Afghanistan, India, Pakistan, Bhutan, Nepal), where outbreaks occur regularly, and some have speculated that this distribution is related to that of the Asian water buffalo (*Bubalus bubalis*). The serotype has been more sporadically reported from countries to the west or east; it has spread periodically into the Middle East and occasionally to Europe (*10*–*13*), but it has not been reported from Africa or the Americas. However, even in its endemic heartland, the Asia 1 serotype has normally been the cause of only a small proportion of cases compared with the proportion caused by serotypes O and A. For example, a study that reviewed FMDV in the West Bengal region of India described recovery of Asia 1 from only 15% of FMD cases examined between 1985 and 2002 (*14*). Similarly, in Southeast Asia, where serotypes O and A are prevalent every year, outbreaks due to Asia 1 have been reported only sporadically in the past 10 years; a recent gap in reporting occurred between 2002 and 2005 (Table; Technical Appendix Table 1).

**Table:** Countries that have reported outbreaks of foot-and-mouth disease virus serotype Asia 1, 2000–2008*

Country	2000	2001	2002	2003	2004	2005	2006	2007	2008
India	x	x	x	x	x	x	x	x	x
Pakistan		x	x	x	x	x			
Iran	x	x	x	x	x	x			
Nepal	x	x	x	x	x		x	x	
Bhutan			x						
Tajikistan					x				
Kyrgyzstan					x				
Afghanistan		x			x				
Turkey	x	x	x						
Myanmar	x	x				x			
Laos		x							
Thailand		x							
Vietnam						x	x	x	
People’s Republic of China						x	x	x	x
Hong Kong						x			
Mongolia						x			
North Korea								x	
Russian Federation						x	x		

*Since 2005, countries are required to report a change in their foot-and-mouth epidemiologic situation only to the World Organisation for Animal Health.

During 2004, evidence showed possible northward spread of the Asia 1 serotype; outbreaks were reported in Kyrgyzstan and Tajikistan. In early 2005, an outbreak was recorded in Hong Kong Special Administrative Region, People’s Republic of China, which suggested that the virus might have crossed China. Later in 2005 and 2006, outbreaks of FMD Asia 1 were reported in several provinces and autonomous regions of China and in Mongolia and Eastern Russia (*15*). In 2005 and 2006, this serotype reappeared in Southeast Asia (Vietnam and Myanmar). This apparent upsurge in cases across a wide geographic area (Figure 1; Technical Appendix Table 1) prompted the current collaborative study to determine the relationships between viruses, with the goal of better understanding the origin of these Asia 1 disease outbreaks.

**Figure 1:**
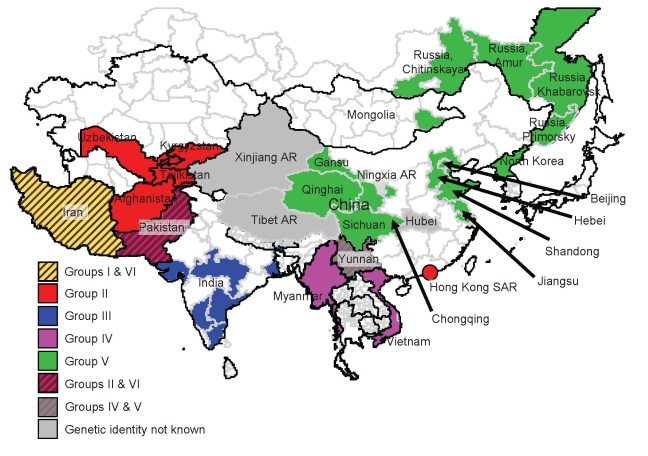
Origin (country and/or region) of isolates of foot-and-mouth disease virus serotype Asia 1 that were responsible for outbreaks in Asia during 2003–2007. The 6 different groups and their localities are indicated by different colors. AR, Autonomous Region; SAR, Special Administrative Region.

## Materials and Methods

### Viruses

Clinical samples containing FMDV Asia 1 were received from Afghanistan, China, Hong Kong, Iran, Kyrgyzstan, Mongolia, Myanmar, Pakistan, Russia, and Tajikistan by the Food and Agriculture Organisation World Reference Laboratory for FMD (WRLFMD), FGI All-Russian Research Institute for Animal Health (Russian Federation), Lanzhou Veterinary Research Institute (China), Project Directorate on FMD (India), Plum Island Animal Disease Center (USA), and Pakchong Regional Reference Laboratory for FMD (Thailand) (Technical Appendix Table 2).

### RNA Extraction, Reverse Transcription–PCR, and DNA Sequencing

RNA extraction, 1-step reverse transcription­–PCR (RT-PCR), and DNA sequencing were performed as previously described (*6*), except that the primer annealing temperature in the RT-PCR was 55°C. The primers used for RT-PCR and DNA sequencing are listed in online Technical Appendix Table 3. Specific methods used by each laboratory can be obtained on request.

### Phylogenetic Analysis

Sequences of these viruses were compared with complete VP1 sequences of Asia 1 viruses stored in the WRLFMD database (n = 300) that have previously been published (*10*,*16*–*18*) or published in this article. Complete VP1 sequences were used to construct a midpoint-rooted neighbor-joining tree using the Kimura 2-parameter nucleotide substitution model as implemented in the program MEGA 4.0 (*19*). The robustness of the tree topology was assessed with 1,000 bootstrap replicates as implemented within the program. The topography of this tree was also checked by the maximum-parsimony (MEGA 4.0) and maximum-likelihood (TREE-PUZZLE 5.2) (*20*) methods, including a selection of isolates from each group to check the robustness of the topography. Subsequently, the sequences were ordered, based on their position in the neighbor-joining phylogenetic tree, and a matrix of percentage nucleotide differences was constructed by using MEGA 4.0. The matrix was imported into Excel 2007 (Microsoft Corporation, Redmond, WA, USA), and conditional formatting was used to identify relationships between sequences in the ranges 95%–100% and 90%–94.9%. The former value was used to group the most closely related virus sequences.

## Results

**Figure 2:**
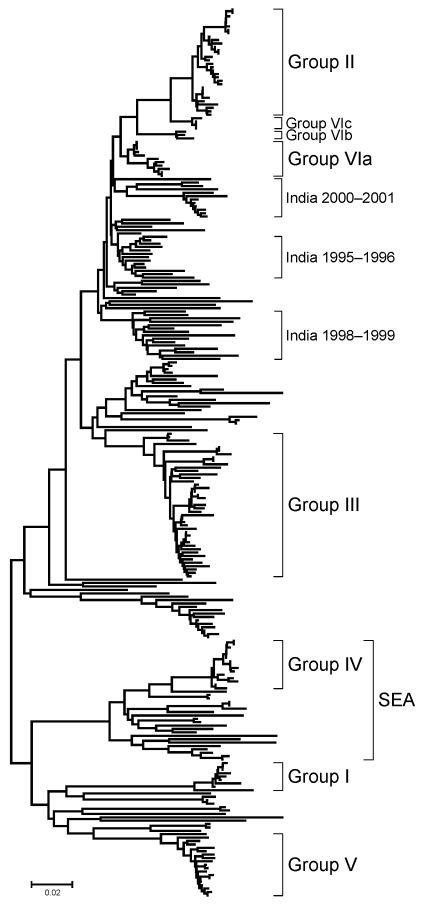
Midpoint-rooted neighbor-joining tree showing the relationships between the complete VP1 sequences of Asia 1 foot-and-mouth disease virus isolates studied. Only the tree structure is shown; details of the labeled groups are given in Figure 3. Scale bar indicates nucleotide substitutions per site. The complete tree with all viruses labeled is shown in the Technical Appendix. SEA, group of viruses found in only in Southeast Asia and Hong Kong.

**Figure 3:**
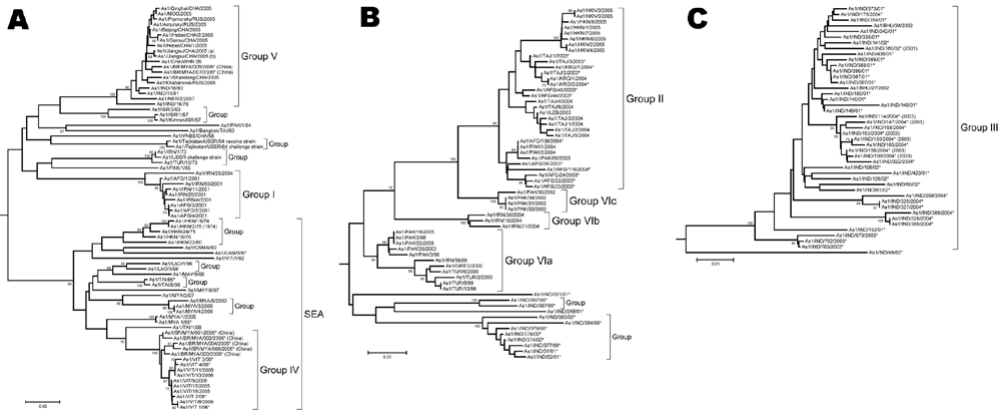
Midpoint-rooted neighbor-joining tree showing relationships between the foot-and-mouth disease Asia 1 viruses studied. A) groups I, IV, and V; B) groups II and VI; C) group III. Other groups of older (pre-2003) viruses sharing >90% nucleotide identity are indicated by the word “group” without any number. Only bootstrap values >70% are shown. Scale bars indicate nucleotide substitutions per site. SEA, group of viruses found in only in Southeast Asia and Hong Kong. *Indicates that the reference number is not one designated by the World Reference Laboratory for Foot-and-Mouth Disease.

The phylogenetic analysis of the complete VP1 gene sequences from isolates of serotype Asia 1 characterized in this study showed that recent viruses (isolated during 2003–2007) belonged to 6 different groups (I–VI) (Figure 2; Technical Appendix). These groups were defined by members of a group having 95%–100% nucleotide identity (Technical Appendix). All groups were supported by bootstrap values of 80%–100% (Technical Appendix) and were found by using alternative phylogenetic algorithms (maximum parsimony and maximum likelihood) (data not shown). Most virus groups were monophyletic. However, 1 group (VI) fell into 3 distinct lineages (a, b, c) and appeared to be ancestral to group II viruses (Figure 3, panel B). This grouping was also evident from the percentage identity matrix, in which the values between viruses in group VI and those in group II were 91.8%–95.9% (Technical Appendix). Relationships between groups II, III, and VI and between group IV and some unnumbered groups were also evident (Technical Appendix).

Viruses that were circulating in Iran in 2004 belonged to 2 different groups (I and VI) (Figures 3, panels A and B). One isolate in group I, collected in Iran in 2004 (IRN/25/2004), was closely related to 8 viruses collected in Afghanistan and Iran in 2001. Other isolates collected in Iran during 2004 belonged to group VIb (e.g., IRN/30/2004) and had <7% nucleotide differences with isolates in group II that were collected in Uzbekistan (2003), Tajikistan (2003–2004), Afghanistan (2004), Kyrgyzstan (2004), Hong Kong (2005), and Pakistan (2002–2004). The report of FMDV Asia 1 in Hong Kong in 2005 was the first since 1980. Notably, the viruses collected in Uzbekistan, Tajikistan, Kyrgyzstan, and Hong Kong in 2003–2005 had <3% nucleotide differences, which suggests that the outbreaks were closely connected and that this virus may have spread a long distance in a short period; however, how this occurred remains unexplained.

Similarly, other viruses collected from Pakistan in 1998, 2003, and 2005 (group VIa) were closely related to viruses responsible for outbreaks in Iran (IRN/58/99), Turkey (TUR/3/2000 and TUR/6/2000), Armenia, Greece (GRE/2/2000), and Georgia from 1999 through 2001 (Figure 3, panel B) and from partial VP1 sequences (data not shown) (*10*,*12*). These data suggest that this epidemic may have originated in Pakistan. Previously, Asia 1 epidemics occurred in 1973 and 1983–1985. In 1973, the virus spread through Iran and Turkey without any traceable origin (*10*) (Figure 3, panel A), and in 1983–1985, genetically closely related viruses were found in many Middle Eastern countries, including Armenia, Azerbaijan, Bahrain, Georgia, Greece, Israel, and Lebanon (represented in the Technical Appendix Figure 1 by LEB/83 and GRE/1/84). However, the ultimate source of this virus strain was also not established (*4*,*10*). Surprisingly, FMD isolates collected in Pakistan in 2003 and 2005 (group VIa) were closely related to PAK/2/98, which had been isolated 5–7 years earlier, with 0.3% and 0.0% nucleotide differences, respectively (Figure 3, panel B). These differences would be consistent with a laboratory escape, use of an improperly inactivated vaccine, or laboratory contamination.

Group III contained only viruses that were collected in India during 2001–2004 and Bhutan (n = 2) during 2002 (Figure 3, panel C). Many other older virus lineages were evident in the phylogenetic analysis (Technical Appendix), showing the diversity of Asia 1 viruses in India. However, most of these lineages have not been detected outside the region, which suggests that endemic Asia 1 viruses rarely spread outside the Indian subcontinent. The reason is not understood.

Within group IV (Figure 3, panel A), FMD Asia 1 viruses responsible for outbreaks in China (Yunnan Province) and Vietnam in 2005 and 2006 were related to viruses originating from Southeast Asia that were collected in Thailand in 1998 and Myanmar in 2005. Viruses in group IV belonged to a larger, more diverse, group of viruses that were found in only in Southeast Asia and Hong Kong from 1974 through 2006 (indicated in Figures 2 and 3, panel A, as SEA). Only 2 viruses originating from Southeast Asia fell outside this supergroup, Bangkok/Thailand/60 (an old vaccine virus strain) and MYA/2/2001 (Technical Appendix). The latter virus clustered with Indian virus isolates, suggesting a possible introduction into Myanmar from the west. In addition, in Myamar, several viruses belonging to 2 sublineages of group IV were detected in a relatively short period (1997–2000 and 2005; Figure 3, panel A), which implies that either multiple lineages are present or that multiple introductions have been made into that country.

FMDV isolates collected in different places in China, the Russian Federation, and Mongolia, during 2005–2006 (group V) were different from viruses isolated in Hong Kong in 2005 (group II) with 16.1%–17.2% nucleotide difference. Another virus belonging to group V has recently (2007) been identified as causing an outbreak of FMD in North Korea (NKR/2/2007) (Figure 3, panel A). The disease likely was introduced by importation of live calves from Liaoning Province, China. Of the 461 susceptible cattle, 431 (≈93%) were infected. All 461 susceptible cattle were destroyed. No cases were exhibited in swine, but 2,630 susceptible swine were destroyed (*21*). Viruses collected in the different provinces or regions of China, Russia, Mongolia, and North Korea during 2005–2007 were closely related to older viruses from India (Tamil Nadu) collected in 1976 and 1980–1981. The nucleotide differences between the Indian viruses and those from China, Mongolia, Russia, and North Korea (Figure 3, panel A) were 0.8%–4.6%, yet the viruses differed markedly from those that were collected more recently in India (group III; Figure 3, panel C) during 2003–2004 (n = 20); nucleotide difference was 12.8%–14.7%. No explanation is readily available, and further investigations need to be performed to determine the origin of the virus responsible for the outbreaks in China. Recently, 7 complete VP1 sequences of Asia 1 FMDV, originating from samples taken from cattle in 2006 in Yunnan Province close to the Myanmar border, were deposited in the public databases (accession nos. EU091342–EU091348; W. Zhang, Y. Hu, F. Zhang, unpub. data). An additional VP1 sequence from a virus from pigs in Sichuan Province in 2006 was also deposited (accession no. EU887277; H. Wang, X. Yang, H. Luo, unpub. data). Five of these sequences belonged to group IV and 2 belonged to group V (Figure, panel A), indicating movements of viruses between China and Southeast Asia and the presence of group V viruses in a more southerly distribution than has previously been reported.

## Discussion

This phylogenetic study demonstrates that the viruses from groups II and V that have been responsible for FMD outbreaks in China appear to have spread large distances in a short time, although the means is unknown. The possibility of spread of viruses of these 2 groups beyond the border where they have been detected must be considered as a potential risk. Furthermore, the close relationships between some recent and older isolates within group V (India 1976–1981 vs. China/Mongolia/Russia/North Korea 2005–2007) and group VIa (Pakistan 1998 vs. Pakistan 2003–2005) raises the question of their origins, either as a result of an unusually slow evolutionary rate or as reintroductions of laboratory/vaccine virus strains.

In Asia, vaccination against FMD varies from country to country; generally, only cattle and water buffalo are vaccinated. Various vaccine strains are used in the region, and vaccines are produced either by large pharmaceutical companies or by national or regional FMD vaccine laboratories. Vaccine matching studies are performed in various FMD reference laboratories on an ad hoc basis, and reference reagents for all the vaccine strains are not always available. This situation requires improvement.

These studies suggest rapid spread of FMD viruses across Asia, but the means by which the viruses are moved has rarely been determined. The spread of some of these FMDV Asia 1 lineages across large parts of Asia, and occasionally outside Asia, demonstrates the continuing need for active surveillance to be improved in Asia to provide real-time monitoring of virus evolution and to disclose more effectively the links between outbreaks. The means of virus transport needs also to be defined, taking into consideration the role played by large antelope populations in central Asia. This information is needed as a prerequisite for further development of regional control programs. India, Pakistan, and China, with their large livestock populations, are expected to play a major role in FMD control in this part of the world.

## Supplementary Material

Technical Appendix Multiple Origins of Foot-and-Mouth Disease Virus Serotype Asia 1 Outbreaks, 2003-2007
