# Supplementary material for: Multiple Origins of Foot-and-Mouth Disease Virus Serotype Asia 1 Outbreaks, 2003–2007
Source: Emerg Infect Dis. 2009 Jul;15(7):1046–51. doi: 10.3201/eid1507.081621 (PMC2744236; doi:10.3201/eid1507.081621)
Supplement: Technical Appendix — Multiple Origins of Foot-and-Mouth Disease Virus Serotype Asia 1 Outbreaks, 2003-2007 [file 08-1621_Techapp-s1.pdf]

Multiple Origins of Foot-and-Mouth Disease Virus Serotype Asia 1 Outbreaks, 2003–2007

Technical Appendix

Table 1. Geographic information and virus phylogenetic characterization on outbreaks of FMD type Asia 1 reported to the OIE or detected by FMD reference laboratories between 2003 and 2007*

Country	Location	Date	Phylogenetic group	Information source (comment)
Afghanistan	Ghazni	2004	II	PIADC
Afghanistan	Balkh	2004	II	PIADC
China	Sheung Shui, New Territories, Hong Kong SAR	2005 Mar 9	II	OIE; WRLFMD
China	Wuxi city, Huishan district, Jiangsu Province	2005 Apr 18	V	OIE (1 outbreak)
China	Tai'an city, Daiyue district, Shandong Province	2005 Apr	V	OIE (1 outbreak)
China	Beijing Province	2005 May	V	OIE
China	Xinjiang Autonomous Region	2005 May	NK	OIE
China	Hebei Province	2005 May–June	V	OIE
China	Qinghai Province	2005 Jul	V	OIE
China	Gansu Province	2005 Jul	V	OIE
China	Gansu Province	2005 Dec	NK	OIE
China	Ningxia Autonomous Region	2005 Dec	NK	OIE
China	Qinghai Province	2005 Dec	NK	OIE
China	Shandong Province	2005 Dec	NK	OIE
China	Jiangsu Province	2005 Dec	NK	OIE
China	Hubei Province	2006 May	V	OIE
China	Gansu Province	2006 May–Jul	NK	OIE
China	Qinghai Province	2006 Jul-Sep	NK	OIE
China	Tibet Autonomous Region	2006 Sep	NK	OIE
China	Yunnan Province	2006	IV, V	Centre for Animal Disease Control and Prevention of Yunnan Province
China	Chongqing, Sichuan Province	2006 Nov	V	OIE
China	Gansu Province	2006 Nov	NK	OIE
China	Xinhe, Xinjiang Province	2007 Jan 23	NK	OIE (subclinical infection)
China	Jintai Agri-Animal Industry Group, Jintai, Gansu Province	2007 Jan 28	V	OIE (clinical disease)
China	Qinglin, Datong, Qinghai Province	2007 Feb 8	NK	OIE (clinical disease)
China	Nalong, Huangyuan, Qinghai Province	2007 Feb 25	NK	OIE (clinical disease)
China	Nalong, Huangyuan, Qinghai Province	2007 May 12	NK	OIE (clinical disease)
China	Duona, Yushu prefecture, Qinghai Province	2007 Jun 7	NK	OIE (107 cattle plus 50 yaks were infected and showed clinical signs)
China	Jiaqiao, Qinghai Province	2007 Oct 24	NK	OIE (clinical disease)
China	Qukou, Pinglao, Ningxia Hui Autonomous Region	2008 Mar 12	NK	OIE (1 outbreak)
China	Qiangganyu, Gangu, Tianshui, Gansu Province	2008 Nov 6	NK	OIE (1 outbreak)
India	Andhra Pradesh, Gujarat, Karnataka, Madhya Pradesh, Tamil Nadu, Tripura, West Bengal	2003–2004	III	PD-FMD
Iran	Damshahr, Qom, Qom	2004 Sep 28	II	WRLFMD
Iran	Oromieh, West Azerbaijan	2004 Nov 8	I	WRLFMD
Iran	Not known	2004	II	WRLFMD
Kyrgyzstan	Not known	2004	II	WRLFMD/ARRIAH

Country	Location	Date	Phylogenetic group	Information source (comment)
Myanmar	Demaw-Hsoe, Loikaw, Kayah	2005 Jul 27	IV	OIE
Myanmar	Saw, Gantgaw, Magway	2005 Nov 22	NK	OIE
Mongolia	Dornod	2005 Aug	V	OIE; ARRIAH
North Korea	Pyongyang	2007	V	WRLFMD
Pakistan	Not known	2003 Oct 9	II	WRLFMD
Pakistan	Lahore, Punjab	2003	VI	WRLFMD
Pakistan	Not known	2004	II	WRLFMD
Pakistan	Lahore and Kharian, Punjab	2005 Jan–Feb	VI	WRLFMD
Russia	Chitinskaya	2006 Jan	V	OIE; ARRIAH
Russia	Amur	2005 Jun	V	OIE; ARRIAH
Russia	Amur	2005 Dec	V	OIE; ARRIAH
Russia	Khabarovsk	2005 Aug	V	OIE; ARRIAH
Russia	Khabarovsk	2005 Dec	V	OIE; ARRIAH
Russia	Primorsky	2005 Sep	V	OIE; ARRIAH
Tajikistan	Khatlonsky region, Vakhdatsky region, Gissarsky region	2003	II	ARRIAH
Tajikistan	NK	2004	II	ARRIAH
Uzbekistan	NK	2003	II	ARRIAH
Vietnam	Van Ninh, Khanh Hoa	2005 Oct 10	IV	OIE
Vietnam	Si Ma Cai, Lao Cai	2005 Oct 13	IV	OIE
Vietnam	NK	2006 May	IV	NK
Vietnam	Various, Quang Tri	2007 Jun 11–30	NK	OIE
Vietnam	Hoang Phuc, Hoang Hoa, Thanh Hoa	2007 Jun 22	NK	OIE

*FMD, foot-and-mouth disease; OIE, World Organisation for Animal Health; PIADC, Plum Island Animal Disease Center; SAR, Special Administrative Region; WRLFMD, Food and Agriculture Organisation World Reference Laboratory for Foot-and-Mouth Disease; NK, not known; CADCP, Centre for Animal Disease Control and Prevention of Yunnan Province; PD-FMD, Project Directorate on Foot-and-Mouth Disease; ARRIAH, All-Russian Institute for Animal Health.

Table 2. The designations, origins, sequence accession numbers and references for the FMD viruses studied

Reference no.	Geographic origin	Country	Date collected	Host species	Laboratory	Accession no.	Reference
AFG/1/2001	Dand, Kandahar	Afghanistan	2001 Feb 8	Bovine	WRLFMD	DQ121109	(*1*)
AFG/2/2001	Dand, Kandahar	Afghanistan	2001 Feb 10	Bovine	WRLFMD	FJ785226	This study
AFG/3/2001	Dand, Kandahar	Afghanistan	2001 Feb 10	Bovine	WRLFMD	FJ785227	This study
AFG/4/2001	Dand, Kandahar	Afghanistan	2001 Feb 10	Bovine	WRLFMD	DQ121110	(*1*)
AFG/22/2003†	Nangahar	Afghanistan	2003 Dec	Bovine	PIADC	EF457987	(*2*)
AFG/24/2003†	Nangahar	Afghanistan	2003 Dec	Bovine	PIADC	EF457988	(*2*)
AFG/26/2003†	Balkh	Afghanistan	2003	Bovine	PIADC	EF457989	(*2*)
AFG/33/2003†	Nangahar	Afghanistan	2003 Dec 17	Bovine	PIADC	EF457990	(*2*)
AFG/40/2003†	Kapisa	Afghanistan	2003 Dec 29	Bovine	PIADC	EF457991	(*2*)
AFG/44/2003†	Kapisa	Afghanistan	2003 Dec 29	Bovine	PIADC	EF457992	(*2*)
AFG/138/2004†	Ghazni	Afghanistan	2004 Feb 13	Bovine	PIADC	EF457994	(*2*)
AFG/116/2004†	Balkh	Afghanistan	2004 Mar 17	Bovine	PIADC	EF457993	(*2*)
BAN/5/87	Duthia, Rajshami, Rajshami	Bangladesh	1987 Feb 14	Bovine	BFAV	NA	(*3*)
BAN/2/96	Savar	Bangladesh	1996 Feb 3	NK	BFAV	NA	(*3*)
BAN/4/96	Savar	Bangladesh	1996 Oct 25	NK	BFAV	NA	(*3*)
BHU/27/2002	Chhokha	Bhutan	2002 Feb 4	NK	WRLFMD	DQ121111	(*1*)
BHU/34/2002	Paro	Bhutan	2002 Feb 12	NK	WRLFMD	DQ121112	(*1*)
CAM/9/80	Tuk Vil, Siem Reap	Cambodia	1980 Nov 27	NK	WRLFMD	FJ785228	This study
CAM/5/97	Chun Lease Dey, Krar Lagn, Siem Reap	Cambodia	1997 Jun 20	Bovine	WRLFMD	FJ785229	This study
YNBS/CHA/58	Yunnan province	China	1958	Bovine	LVRI	AY390432	Chang et al., unpub. data
Jiangsu/CHA/2005 (a)	Jiangsu Province	China	2005 Apr	Bovine	LVRI	DQ156527	(*1*)
Jiangsu/CHA/2005 (b)	Wuxi city, Huishan district, Jiangsu province	China	2005 Apr	Bovine	LVRI	EF149009	This study
Beijing/CHA/2005	Beijing municipality, Yanqing county	China	2005 May	Bovine	LVRI	EF185303	This study
HeB/CHA/2/2005	Sanhe city, Hebei province	China	2005 May	Bovine	LVRI	EF187273	This study
HeB/CHA/1/2005	Zhangjiakou city, Hebei province	China	2005 Jun	Bovine	LVRI	EF187274	This study
JingNing/GS/CHA/2005	Pingliang city, Jingning county, Gansu province	China	2005 Jul	Bovine	LVRI	EF185304	This study
TongRen/QH/CHA/2005	Huangnan district, Tongren county, Qinghai province	China	2005 Jul	Bovine	LVRI	EF187272	This study
BR/MYA/001/2006†	Yunnan Province (border region with Myanmar)	China	2006	Bovine	CADCP	EU091342	Zhang et al., unpub. data
BR/MYA/002/2006†	Yunnan Province (border region with Myanmar)	China	2006	Bovine	CADCP	EU091343	Zhang et al., unpub. data
BR/MYA/003/2006†	Yunnan Province (border region with Myanmar)	China	2006	Bovine	CADCP	EU091344	Zhang et al., unpub. data
BR/MYA/004/2006†	Yunnan Province (border region with Myanmar)	China	2006	Bovine	CADCP	EU091345	Zhang et al., unpub. data
BR/MYA/005/2006†	Yunnan Province (border region with Myanmar)	China	2006	Bovine	CADCP	EU091346	Zhang et al., unpub. data
BR/MYA/006/2006†	Yunnan Province (border region with Myanmar)	China	2006	Bovine	CADCP	EU091347	Zhang et al., unpub. data
BR/MYA/007/2006†	Yunnan Province (border region with Myanmar)	China	2006	Bovine	CADCP	EU091348	Zhang et al., unpub. data
CHA/WHN 06†	Sichuan Province	China	2006	Porcine	Sichuan Univ.	EU887277	Wang et al., unpub. data
GRE/1/84	Evros	Greece	1984	Bovine	WRLFMD	EU553909	(*4*)
GRE/2/2000	Feres, Evros	Greece	2000 Jul 1	NK	WRLFMD	DQ121113	(*1*)

Reference no.	Geographic origin	Country	Date collected	Host species	Laboratory	Accession no.	Reference
HKN/19/74	Ping Yeung, San Tsuen, Takwuling, NT, Kowloon	Hong Kong	1974 Oct 3	Bovine	WRLFMD	FJ785230	This study
HKN/2/75 (1974)	Ping Che, Takwuling, NT, Kowloon	Hong Kong	1974 Dec 23	Bovine	WRLFMD	FJ785231	This study
HKN/24/75	Dairy Farm, Pokfulam Road, Hong Kong Island	Hong Kong	1975 Feb 15	Bovine	WRLFMD	FJ785232	This study
HKN/18/76	Wong Ka Wai, Tung Chung, Lantau Island	Hong Kong	1976 Feb 10	Bovine	WRLFMD	FJ785233	This study
HKN/22/80	Shek Po Tsuen, Hing Shui Kui, Ping Shan, Yuen Long	Hong Kong	1980 Aug 23	Bovine	WRLFMD	FJ785234	This study
HKN/1/2005	Sheung Shui, New Territories	Hong Kong	2005 Mar 9	Bovine	WRLFMD	DQ121114	(*1*)
HKN/2/2005	Sheung Shui, New Territories	Hong Kong	2005 Mar 9	Bovine	WRLFMD	DQ121115	(*1*)
HKN/3/2005	Sheung Shui, New Territories	Hong Kong	2005 Mar 9	Bovine	WRLFMD	FJ785235	This study
HKN/4/2005	Sheung Shui, New Territories	Hong Kong	2005 Mar 9	Bovine	WRLFMD	FJ785236	This study
HKN/5/2005	Sheung Shui, New Territories	Hong Kong	2005 Mar 10	Bovine	WRLFMD	FJ785237	This study
HKN/6/2005	Sheung Shui, New Territories	Hong Kong	2005 Mar 10	Bovine	WRLFMD	FJ785238	This study
HKN/7/2005	Sheung Shui, New Territories	Hong Kong	2005 Mar 10	Bovine	WRLFMD	FJ785239	This study
HKN/8/2005	Sheung Shui, New Territories	Hong Kong	2005 Mar 10	Bovine	WRLFMD	FJ785240	This study
IND/2/71 (1964)	Mukteswar Dairy	India	1964 Feb 25	Bovine	WRLFMD	FJ785241	This study
IND/63/72†	Maharashtra	India	1972	Bovine	other	Y09949	(*5*)
IND/16/76	Ranipet, Vellore District, Tamil Nadu	India	1976	NK	WRLFMD	FJ785242	This study
IND/8/79	NK	India	1979	NK	WRLFMD	EU553910	(*4*)
IND/18/80	Kargudy, Tamil Nadu	India	1980 Sep 20	Bovine	WRLFMD	DQ121116	(*1*)
IND/15/81	Mandakarai, Nilgiris, Tamil Nadu	India	1981 Feb 17	Bovine	WRLFMD	DQ121117	(*1*)
WBN/117/85†	Nadia, West Bengal	India	1985	NK	WRLFMD	EU553911	(*4*)
IND/WBN/117/85† (IND 491/97†)	Nadia, West Bengal	India	1985	NK	PD-FMD	AY687334	(*6*)
IND/WBN/117/85† (IND 491/97†)	Nadia, West Bengal	India	1985	NK	PD-FMD	AF392922	(*7*)
IND 4/86†	NK	Bhutan	1986	Bovine	PD-FMD	AF392944	Sanyal et al., unpub. data
IND 75/86†	Assam	India	1985 Oct 5	Bovine	PD-FMD	AF390702	(*7*)
IND 46/87†	Rajasthan	India	1986 Feb 14	Bovine	PD-FMD	AF390692	(*7*)
IND 22/88†	Assam	India	1987 Sep 28	Bovine	PD-FMD	AF390685	(*7*)
IND 120/88†	Maharashtra	India	1988 Mar 24	Bovine	PD-FMD	AF390675	(*7*)
IND 177/88†	Maharashtra	India	1988 Jan 19	Bovine	PD-FMD	AF392904	(*7*)
IND 19/89†	Uttar Pradesh	India	1989 Apr 29	Buffalo	PD-FMD	AF390684	(*7*)
IND 45/89†	Uttar Pradesh	India	1989	Buffalo	PD-FMD	AF390691	(*7*)
IND 2/90†	Uttar Pradesh	India	1990 Jan 6	Bovine	PD-FMD	AF392912	(*7*)
IND 116/90†	Haryana	India	1990	Bovine	PD-FMD	AF392926	Sanyal et al., unpub. data
IND 132/90†	Uttar Pradesh	India	1990 Apr 26	Bovine	PD-FMD	AF390676	(*7*)
IND 13/91†	Maharashtra	India	1990 Sep 21	Ovine	PD-FMD	AF390677	(*7*)
IND 17/91†	Maharashtra	India	1990 Sep 11	Porcine	PD-FMD	AF390682	(*7*)
IND 68/92†	Andhra Pradesh	India	1992	Bovine	PD-FMD	AF392946	Sanyal et al., unpub. data
IND 247/92†	Maharashtra	India	1992	Bovine	PD-FMD	AF392932	Sanyal et al., unpub. data
IND 46/93†	Uttar Pradesh	India	1993 Feb 17	Bovine	PD-FMD	TBS	(*8*)
IND 49/93†	Uttar Pradesh	India	1993 Feb 22	Buffalo	PD-FMD	AF392945	(*8*)

Reference no.	Geographic origin	Country	Date collected	Host species	Laboratory	Accession no.	Reference
IND 53/93†	Karnataka	India	1993 Mar 15	Bovine	PD-FMD	AF390695	(*7*)
IND 152/94†	Haryana	India	1993 Dec 16	Bovine	PD-FMD	AF392930	(*8*)
IND 175/94†	Karnataka	India	1994 Jul 21	Bovine	PD-FMD	AF392931	(*8*)
IND 298/94†	Uttar Pradesh	India	1994	Bovine	PD-FMD	AF392935	Sanyal et al., unpub. data
IND 305/94†	Andhra Pradesh	India	1994	Bovine	PD-FMD	AF392936	(*8*)
IND 1/95†	Karnataka	India	1994 Sep 14	Bovine	PD-FMD	AF390683	(*7*)
IND 6/95†	Karnataka	India	1994 Sep 7	Bovine	PD-FMD	AF390697	(*7*)
IND 14/95†	Andhra Pradesh	India	1995	Bovine	PD-FMD	AF390678	(*7*)
IND 24/95†	Karnataka	India	1994 Dec 22	Bovine	PD-FMD	AF392933	(*8*)
IND 26/95†	Uttar Pradesh	India	1995	Bovine	PD-FMD	AF390687	(*7*)
IND 36/95†	Tamil Nadu	India	1995 Dec 17	Bovine	PD-FMD	TBS	(*8*)
IND 47/95†	Andhra Pradesh	India	1995	Bovine	PD-FMD	AF392921	(*7*)
IND 50/95†	Uttar Pradesh	India	1995	Buffalo	PD-FMD	AF390694	(*7*)
IND 57/95†	Andhra Pradesh	India	1995	Bovine	PD-FMD	AF390696	(*7*)
IND 33/96†	West Bengal	India	1995 Dec 6	Bovine	PD-FMD	AF390689	(*7*)
IND 43/96†	Maharashtra	India	1996 Feb 8	Bovine	PD-FMD	AF390690	(*7*)
IND 72/96†	Maharashtra	India	1995	Bovine	PD-FMD	AF390700	(*7*)
IND 73/96†	Pondichery	India	1995	Bovine	PD-FMD	AF390701	(*7*)
IND 80/96†	Karnataka	India	1996 Mar 12	Bovine	PD-FMD	AF390703	(*7*)
IND 81/96†	Karnataka	India	1996 Mar 16	Bovine	PD-FMD	AF390704	(*7*)
IND 82/96†	Himachal Pradesh	India	1996	Bovine	PD-FMD	AF390705	(*7*)
IND 89/96†	Punjab	India	1996 Mar 29	Buffalo	PD-FMD	AF390706	(*7*)
IND 173/96†	Haryana	India	1996 Jun 6	Buffalo	PD-FMD	AF390681	(*7*)
IND 339/96†	Kerala	India	1996	Bovine	PD-FMD	AF392939	Sanyal et al., unpub. data
IND 256/97†	Tamilnadu	India	1997	Bovine	PD-FMD	AF392906	(*7*)
IND 386/97†	Karnataka	India	1994 May 19	Bovine	PD-FMD	AF392914	(*7*)
IND 388/97†	Karnataka	India	1994 Jul 4	Bovine	PD-FMD	AF392915	(*7*)
IND 390/97†	Karnataka	India	1994 Jul 13	Bovine	PD-FMD	AF392940	(*8*)
IND 396/97†	Karnataka	India	1994 Sep 28	Bovine	PD-FMD	AF392941	(*8*)
IND 397/97†	Karnataka	India	1994 Nov 3	Bovine	PD-FMD	AF392942	(*8*)
IND 44/98†	Maharashtra	India	1998	Bovine	PD-FMD	AF392943	(*8*)
IND 125/98†	Orissa	India	1998 Jan 8	Bovine	PD-FMD	AF392928	(*8*)
IND 270/98†	West Bengal	India	1998 Mar 25	Bovine	PD-FMD	AF392907	(*7*)
IND 271/98†	West Bengal	India	1998 Apr 16	Bovine	PD-FMD	AF392908	(*7*)
IND 69/99†	Karnataka	India	1998 Nov 24	Bovine	PD-FMD	AF392947	(*8*)
IND 92/99†	Haryana	India	1998	Bovine	PD-FMD	AF392925	(*7*)
IND 102/99†	Haryana	India	1999 Jan 7	Buffalo	PD-FMD	AF392897	(*7*)
IND 104/99†	Haryana	India	1999 Jan 7	Buffalo	PD-FMD	AF392898	(*7*)
IND 105/99†	Haryana	India	1999	Bovine	PD-FMD	AF392899	(*7*)
IND 107/99†	Punjab	India	1998 Dec 31	Bovine	PD-FMD	AF392900	(*7*)
IND 108/99†	Punjab	India	1999	Bovine	PD-FMD	AF392902	(*7*)
IND 126/99†	Karnataka	India	1998 Dec 17	Bovine	PD-FMD	AF392903	(*7*)
IND 127/99†	Karnataka	India	1998 Dec 17	Bovine	PD-FMD	AF392929	(*8*)
IND 192/99†	West Bengal	India	1999 Jan 24	Bovine	PD-FMD	TBS	(*8*)
IND 196/99†	Karnataka	India	1999 Feb 2	Bovine	PD-FMD	AF392905	(*7*)
IND 235/99†	Haryana	India	1999	Buffalo	PD-FMD	TBS	(*8*)
IND 277/99†	Karnataka	India	1999 Mar 12	Bovine	PD-FMD	AF392909	(*7*)
IND 278/99†	Karnataka	India	1999 Mar 12	Bovine	PD-FMD	AF392934	(*8*)
IND 286/99†	Haryana	India	1999	Bovine	PD-FMD	AF392911	(*7*)
IND 367/00†	West Bengal	India	2000 Oct 14	Bovine	PD-FMD	TBS	(*8*)
IND 374/00†	Karnataka	India	2000	Bovine	PD-FMD	TBS	(*8*)
IND 377/00†	Karnataka	India	2000	Bovine	PD-FMD	TBS	(*8*)
IND 378/00†	Karnataka	India	2000	Bovine	PD-FMD	TBS	(*8*)
IND 379/00†	Karnataka	India	2000	Bovine	PD-FMD	TBS	(*8*)
IND 383/00†	Karnataka	India	2000 Oct 13	Bovine	PD-FMD	TBS	(*8*)
IND 384/00†	Karnataka	India	2000 Oct 17	Bovine	PD-FMD	TBS	(*8*)
IND 387/00†	Karnataka	India	2000 Oct 25	Bovine	PD-FMD	TBS	(*8*)
IND 51/01†	Karnataka	India	2001	Bovine	PD-FMD	TBS	(*8*)
IND 52/01†	Karnataka	India	2001	Bovine	PD-FMD	TBS	(*8*)
IND 107/01†	Himachal Pradesh	India	2001	Bovine	PD-FMD	TBS	(*8*)
IND 140/01†	Gujarat	India	2000 Dec 22	Bovine	PD-FMD	TBS	(*8*)
IND 148/01†	Gujarat	India	2000 Dec 20	Bubaline	PD-FMD	TBS	(*8*)

Reference no.	Geographic origin	Country	Date collected	Host species	Laboratory	Accession no.	Reference
IND 149/01†	Gujarat	India	2001 Jan 10	Bovine	PD-FMD	TBS	(*8*)
IND 152/01†	Gujarat	India	2001 Jan 20	Bovine	PD-FMD	TBS	(*8*)
IND 160/01†	Gujarat	India	2001 Feb 23	Bovine	PD-FMD	TBS	(*8*)
IND 248/01†	West Bengal	India	2001 Mar 8	Bovine	PD-FMD	TBS	(*8*)
IND 321/01†	Madhya Pradesh	India	2001 Aug	Bovine	PD-FMD	AY687333	(*6*)
IND 335/01†	Uttar Pradesh	India	2001 Sep 15	Bovine	PD-FMD	TBS	(*8*)
IND 342/01†	Bihar	India	2001 Aug 22	Bovine	PD-FMD	TBS	(*8*)
IND 354/01†	Assam	India	2001 Sep 28	Bovine	PD-FMD	TBS	(*8*)
IND 373/01†	West Bengal	India	2001 Jul 25	Bovine	PD-FMD	TBS	(*8*)
IND 387/01†	Uttar Pradesh	India	2001 Nov 27	Bovine	PD-FMD	TBS	(*8*)
IND 388/01†	Uttar Pradesh	India	2001 Nov 27	Bovine	PD-FMD	TBS	(*8*)
IND 389/01†	Uttar Pradesh	India	2001 Nov 27	Bovine	PD-FMD	TBS	(*8*)
IND 396/01†	Uttar Pradesh	India	2001 Dec 4	Bovine	PD-FMD	TBS	(*8*)
IND 397/01†	Uttar Pradesh	India	2001 Dec 4	Bovine	PD-FMD	TBS	(*8*)
IND 423/01†	Haryana	India	2001	Bovine	PD-FMD	TBS	(*8*)
IND 438/01†	Uttar Pradesh	India	2001 Dec 11	Bovine	PD-FMD	TBS	(*8*)
IND 60/02†	Gujarat	India	2002	Bovine	PD-FMD	DQ101243	(*1*)
IND 61/02†	Gujarat	India	2002	Bovine	PD-FMD	TBS	(*8*)
IND 139/02†	Bihar	India	2002 Feb 27	Bovine	PD-FMD	DQ101242	(*1*)
IND 141/02†	West Bengal	India	2002 Oct 3	Bovine	PD-FMD	TBS	(*8*)
IND 180/02†	West Bengal	India	2002 Dec 24	Bovine	PD-FMD	FJ785291	This study
IND 198/02†	West Bengal	India	2002	Bovine	PD-FMD	DQ101244	(*1*)
IND 673/2003†	Tamil Nadu	India	2003	Bovine	PD-FMD	DQ101241	(*1*)
IND 762/2003†	Andhra Pradesh	India	2003	Bovine	PD-FMD	DQ101240	(*1*)
IND 763/2003†	Andhra Pradesh	India	2003	Bovine	PD-FMD	FJ785292	This study
IND 4/2004†	Karnataka	India	2003 Nov	NK	PD-FMD	FJ785293	This study
IND 114/2004† (2003)	West Bengal	India	2003 Oct 30	Bovine	PD-FMD	DQ101239	(*1*)
IND 147/2004†	West Bengal	India	2003 Dec 3	Bovine	PD-FMD	FJ785294	This study
IND 150/2004†	West Bengal	India	2003 Dec 9	Bovine	PD-FMD	FJ785295	This study
IND 153/2004†	West Bengal	India	2003 Dec 22	Bovine	PD-FMD	FJ785296	This study
IND 156/2004†	West Bengal	India	2003 Dec 17	Bovine	PD-FMD	FJ785297	This study
IND 158/2004†	West Bengal	India	2003 Dec 24	Bovine	PD-FMD	FJ785298	This study
IND 165/2004†	West Bengal	India	2004 Jan 27	Bovine	PD-FMD	DQ101238	(*1*)
IND 168/2004†	West Bengal	India	2004 Feb 12	Bovine	PD-FMD	FJ785299	This study
IND 175/2004†	Tripura	India	2004	Bovine	PD-FMD	DQ101237	(*1*)
IND 268/2004†	Madhya Pradesh	India	2004 Apr 9	Bovine	PD-FMD	DQ101236	(*1*)
IND 322/2004†	West Bengal	India	2004 Mar 10	Bovine	PD-FMD	FJ785300	This study
IND 325/2004†	West Bengal	India	2004 Mar 18	Bovine	PD-FMD	FJ785301	This study
IND 327/2004†	West Bengal	India	2004 Mar 18	Bovine	PD-FMD	FJ785302	This study
IND 328/2004†	West Bengal	India	2004 Mar 9	Bovine	PD-FMD	FJ785303	This study
IND 388/2004†	Gujarat	India	2004	Bovine	PD-FMD	DQ101235	(*1*)
IND 389/2004†	Gujarat	India	2004	Bovine	PD-FMD	FJ785304	This study
IRN/1/73	NK	Iran	1973	NK	WRLFMD	EU553912	(*4*)
IRN/58/99	Tehran	Iran	1999 Jun 20	NK	WRLFMD	DQ121122	(*1*)
IRN/4/2001	Lov, Mohamad Abad, Esfahn, Mohamad Abad	Iran	2001 Jul 6	Bovine	WRLFMD	DQ121118	(*1*)
IRN/11/2001	Shimiz Abad, Markalay	Iran	2001 Jul 8	Bovine	WRLFMD	FJ785243	This study
IRN/25/2001	NK	Iran	2001	NK	WRLFMD	FJ785244	This study
IRN/63/2001	Takily, Talesh, Gilan	Iran	2001 Dec 17	Bovine	WRLFMD	FJ785245	This study
IRN/10/2004	Damshahr, Qom, Qom	Iran	2004 Sep 28	Bovine	WRLFMD	DQ121119	(*1*)
IRN/25/2004	Oromieh, West Azerbaijan	Iran	2004 Nov 8	Bovine	WRLFMD	DQ121120	(*1*)
IRN/30/2004	NK	Iran	2004	NK	WRLFMD	FJ785246	This study
IRN/31/2004	NK	Iran	2004	NK	WRLFMD	DQ121121	(*1*)
ISR/1/57	NK	Israel	1957	NK	WRLFMD	EU553913	(*4*)
Kimron/ISR/57	NK	Israel	1957	NK	PIADC	AY593797	(*9*)
ISR/3/63	Yokneam	Israel	1963	NK	PIADC	AY593796	(*9*)
Shamir/ISR/89	Shamir	Israel	1989	NK	BFAV	NA	(*3*)
KRG/1/2004	South	Kyrgyzstan	2004	NK	WRLFMD	FJ785247	This study
KRG/1/2004†	NK	Kyrgyzstan	2004	NK	ARRIAH	FJ785248	This study
KRG/2/2004†	NK	Kyrgyzstan	2004	NK	ARRIAH	FJ785249	This study

Reference no.	Geographic origin	Country	Date collected	Host species	Laboratory	Accession no.	Reference
LAO/1/96	Vientiene Municipality	Laos	1996 Jun 12	NK	WRLFMD	EU667460	(*10*)
LAO/3/98	Vientiene Municipality	Laos	1998 Jan 5	Bovine	WRLFMD	EU667461	(*10*)
LEB/83	Kfar Kela	Lebanon	1983 Nov	Bovine	PIADC	AY593799	(*9*)
LEB/83	Kfar Kela	Lebanon	1983 Nov	Bovine	WRLFMD	AJ294931	(*11*)
MAY/8/97	Block 15, Felda Kemamang, Tanah Meran, Sedok	Malaysia	1997 Aug 13	Bovine	WRLFMD	FJ785250	This study
MAY/9/99	Kawasan, Masjid Tanah	Malaysia	1999 Apr 16	Bovine	WRLFMD	FJ785251	This study
MOG/2005†	Dornod	Mongolia	2005 Aug	Bovine	ARRIAH	FJ785252	This study
MYA/2/97	U Kyi Win, Moe Nat Kone, Thayet	Myanmar	1997 Nov 23	Bovine	WRLFMD	FJ785253	This study
MYA/3/2000	U Aung Khin, Kayin Gyaung, Kyauk Taw	Myanmar	2000 Jun 12	Bovine	WRLFMD	FJ785254	This study
MYA/4/2000	U Kan Tun, Pho Kalar, Tike Gti	Myanmar	2000 Jun 21	Bovine	WRLFMD	FJ785255	This study
MYA/5/2000	U Myo Hylaing, Lei Pyin, Tharyarnady	Myanmar	2000 May 11	Bovine	WRLFMD	FJ785256	This study
MYA/2/2001	Uthatun Aye, Tainnyo, Mrauk-u, Rakhine	Myanmar	2001 Jul 13	Bovine	WRLFMD	DQ121123	(*1*)
MYA 1/05†	Kwethawdaw, Demawsoe, Loikaw, Kayah State	Myanmar	2005 Jul 28	NK	TRRL	FJ785257	This study
MYA/1/2005	Kwethawdaw, Demawsoe, Loikaw, Kayah State	Myanmar	2005 Jul 28	NK	WRLFMD	FJ785258	This study
NKR/2/2007	Pyongyang	DPR of Korea	2007	Bovine	WRLFMD	FJ785259	This study
PAK/1/54	Okara, Punjab	Pakistan	1954 Mar 4	Buffalo	PIADC	AY593795	(*9*)
PAK/1/85	Lahore City, Punjab	Pakistan	1985	Bovine	WRLFMD	FJ785260	This study
PAK/2/98	20 Miles from Lahore, Punjab	Pakistan	1998	Bovine	WRLFMD	EU553914	(*4*)
PAK/3/98	NK	Pakistan	1998	Bovine	WRLFMD	FJ785261	This study
PAK/30/2002	NK	Pakistan	2002 Oct 31	Buffalo	WRLFMD	DQ121124	(*1*)
PAK/31/2002	NK	Pakistan	2002 Nov 6	Buffalo	WRLFMD	DQ121125	(*1*)
PAK/33/2002	NK	Pakistan	2002 Nov 6	Buffalo	WRLFMD	FJ785262	This study
PAK/34/2002	NK	Pakistan	2002 Nov 6	Buffalo	WRLFMD	FJ785263	This study
PAK/20/2003	Lahore, Punjab	Pakistan	2003	NK	WRLFMD	DQ121126	(*1*)
PAK/69/2003	NK	Pakistan	2003 Oct 9	Bovine	WRLFMD	DQ121127	(*1*)
PAK/1/2004	NK	Pakistan	2004	NK	WRLFMD	DQ121128	(*1*)
PAK/2/2004	NK	Pakistan	2004	NK	WRLFMD	FJ785264	This study
PAK/19/2005	Lahore, Punjab	Pakistan	2005 Jan 25	Bovine	WRLFMD	FJ785265	This study
PAK/22/2005	Kharian, Punjab	Pakistan	2005 Feb 7	Bovine	WRLFMD	FJ785266	This study
Amursky/RUS/2005	Amursky	Russian Federation	2005	Bovine	ARRIAH	DQ121401	(*1*)
Khabarovsk/RUS/2005	Khabarovsk	Russian Federation	2005	Bovine	ARRIAH	FJ785267	This study
Prymorsky/RUS/2005	Prymorsky	Russian Federation	2005	Bovine	ARRIAH	FJ785268	This study
Bangkok/60	Bangkok	Thailand	1960	NK	WRLFMD	FJ785269	This study
TAI/85†	Petchaburi Province	Thailand	1985	Bovine	WRLFMD	NA	This study
TAI/5/96†	NK	Thailand	1996	NK	BFAV	NA	(*3*)
TAI/1/98	Chiengrai (Chiang Rai)	Thailand	1998 Jan 10	Buffalo	WRLFMD	DQ121129	(*1*)
TAJ/1/2003†	Khatlonsky region	Tajikistan	2003 Oct 11	Bovine	ARRIAH	FJ785270	This study
TAJ/2/2003†	Vakhdatsky region	Tajikistan	2003 Aug 10	Bovine	ARRIAH	FJ785271	This study
TAJ/3/2003†	Gissarsky region	Tajikistan	2003 Mar 10	Bovine	ARRIAH	FJ785272	This study
TAJ/1/2004†	NK	Tajikistan	2004	NK	ARRIAH	DQ121402	(*1*)
TAJ/2/2004†	NK	Tajikistan	2004	NK	ARRIAH	DQ121403	(*1*)
TAJ/3/2004†	NK	Tajikistan	2004	NK	ARRIAH	FJ785273	This study
TAJ/4/2004†	NK	Tajikistan	2004	NK	ARRIAH	FJ785274	This study
TAJ/5/2004†	NK	Tajikistan	2004	NK	ARRIAH	FJ785275	This study
TAJ/6/2004†	NK	Tajikistan	2004	NK	ARRIAH	FJ785276	This study

Reference no.	Geographic origin	Country	Date collected	Host species	Laboratory	Accession no.	Reference
TUR/15/73	Bayrampaşa, Merkez, Kars	Turkey	1973 Aug 12	Bovine	WRLFMD	EU553917	(*4*)
TUR/8/99	Sadikli (Sadakli), Eleskirt, Agri	Turkey	1999 Jan 10	Bovine	WRLFMD	DQ121130	(*1*)
TUR/10/99	Gifteanar (Ciftepcnar), Eleskirt, Agri	Turkey	1999 Jan 10	Bovine	WRLFMD	DQ121131	(*1*)
TUR/3/2000	Tukat/Zile, Kirikkale	Turkey	2000	Bovine	WRLFMD	EU553915	(*4*)
TUR/6/2000	Sivas	Turkey	2000	Bovine	WRLFMD	EU553916	(*4*)
UZB/2003	NK	Uzbekistan	2003	NK	ARRIAH	FJ785277	This study
USSR challenge strain	NK	NK	NK	NK	Other	U87835	(*12*)
Tajikistan/USSR/64 vaccine strain	Tajikistan	USSR	1964	NK	ARRIAH	FJ785278	This study
Tajikistan/USSR/64 challenge strain	Tajikistan	USSR	1964	NK	ARRIAH	FJ785279	This study
VIT/1/92	Binh Ba Bay (202 km from Ho Chi Minh City)	Vietnam	1992 Oct 19	Bovine	WRLFMD	FJ785280	This study
VIT/15/2005	Kanh Hoa	Vietnam	2005 Oct 18	Bovine	WRLFMD	FJ785281	This study
VIT/16/2005	Kanh Hoa	Vietnam	2005 Oct 27	Bovine	WRLFMD	FJ785282	This study
VIT/8/2006	Khanh Hoa Province	Vietnam	2005 Oct 27	Bovine	WRLFMD	FJ785283	This study
VIT 1/06†	Khanh Hoa Province	Vietnam	2005 Oct 27	Bovine	TRRL	FJ785284	This study
VIT/9/2006	Khanh Hoa Province	Vietnam	2005 Oct 27	Bovine	WRLFMD	FJ785285	This study
VIT 2/06†	Khanh Hoa Province	Vietnam	2005 Oct 27	Bovine	TRRL	FJ785286	This study
VIT/10/2006	Lao Cai Province	Vietnam	2005 Nov 11	Buffalo	WRLFMD	FJ785287	This study
VIT 3/06†	Lao Cai Province	Vietnam	2005 Nov 11	Buffalo	TRRL	FJ785288	This study
VIT/11/2006	Lao Cai Province	Vietnam	2005 Nov 11	Buffalo	WRLFMD	FJ785289	This study
VIT 4/06†	Lao Cai Province	Vietnam	2005 Nov 11	Buffalo	TRRL	FJ785290	This study

*FMD, foot-and-mouth disease; WRLFMD, Food and Agriculture Organisation World Reference Laboratory for Foot-and-Mouth Disease; PIADC, Plum Island Animal Disease Center; BFAV, Bundesforschungsanstalt für Viruskrankheiten der Tiere; NA, not available; NK, not known; LVRI, Lanzhou Veterinary Research Institute; CADCP, Centre for Animal Disease Control and Prevention of Yunnan Province; SAR, Special Administrative Region; PD-FMD, Project Directorate on Foot-and-Mouth Disease; TBS, to be submitted; ARRIAH, All-Russian Institute for Animal Health; TRRL, Thailand Regional Reference Laboratory.
†Not a WRLFMD reference number.

Table 3. Oligonucleotide primers used for RT-PCR and cycle sequencing of FMDV*

Primer	Primer sequence (5′ → 3′)	Direction	Location on the FMDV genome		Use
			Gene	Position†	
As1-1C505F	TACACTGCTTCTGACGTGGC	Forward	1C	3066–3085	PCR
As1-1C530F	CCACRAGTGTGCARGGATGGGT	Forward	1C	3091–3112	PCR
As1-1C613F	GCCGGCAARGAYTTTGAGTTYCG	Forward	1C	3174–3196	PCR
As1-1C616F	GGCAAGGACTTTGAGTTTCGC	Forward	1C	3177–3197	Sequencing
As1-1D205F	GCRACGTACTACTTYTCRGACCT	Forward	1D	3423–3445	Sequencing
As1-1D370R	GTTGTAYACTGTYGCCAGCACACG	Reverse	1D	3588–3611	Sequencing
NK72	GAAGGGCCCAGGGTTGGACTC	Reverse	2A/2B	3885–3905	Sequencing
NK61	GACATGTCCTCCTGCATCTG	Reverse	2B	3963–3982	RT-PCR
EUR-2B52R	GACATGTCCTCCTGCATCTGGTTGAT	Reverse	2B	3957–3982	RT-PCR

*RT-PCR, reverse transcription–PCR; FMDV, foot-and-mouth disease viruses;
†On the genome of Asia1/IND/63/72 (EMBL/GenBank accession no. AY304994).

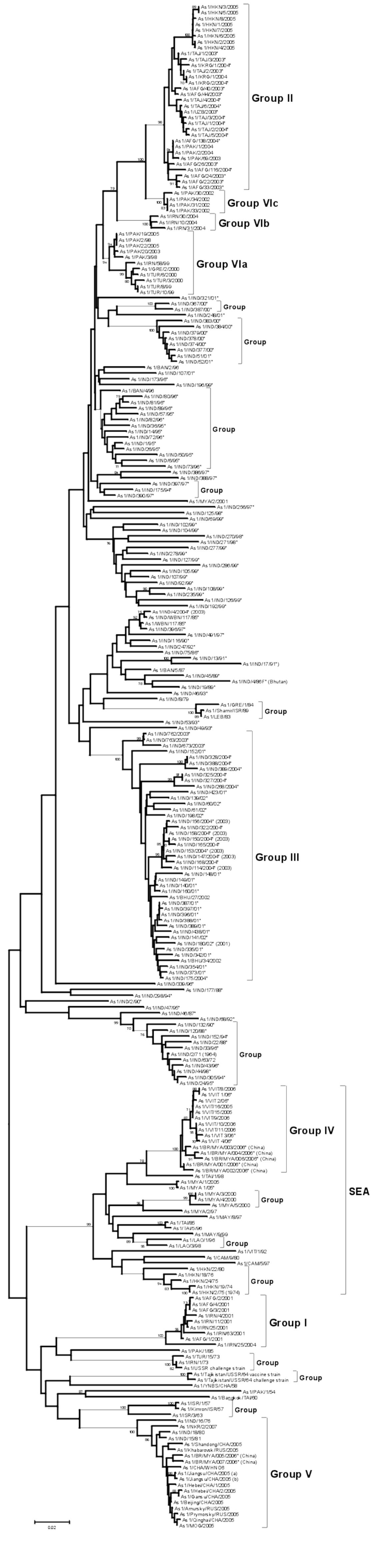

Technical Appendix Figure 1. Midpoint-rooted neighbor-joining tree showing the relationships between the complete VP1 sequences of Asia 1 foot-and-mouth disease virus isolates studied.

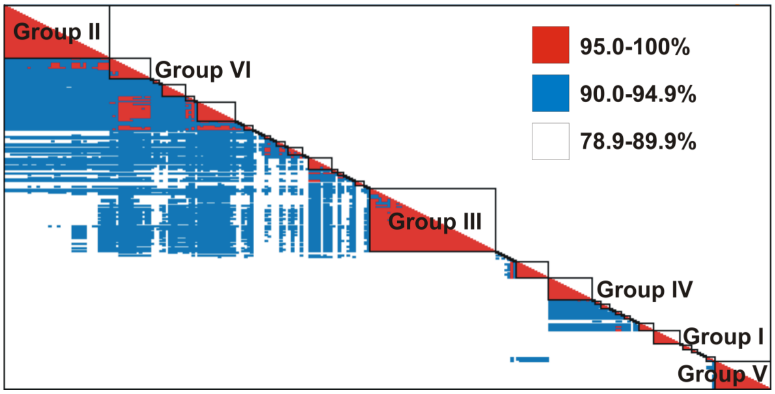

Technical Appendix Figure 2. Percentage nucleotide identity matrix showing the relationships between all the foot-and-mouth disease Asia 1 viruses VP1 sequences examined.

References

1. Valarcher JF, Knowles NJ, Ferris NP, Paton DJ, Zakharov V, Sherbakov A, et al. Recent spread of foot-and-mouth disease virus serotype Asia 1. Vet Rec. 2005;157:30. PubMed

2. Schumann KR, Knowles NJ, Davies PR, Midgley RJ, Valarcher J-F, Raoufi AQ, et al. Genetic characterization and molecular epidemiology of foot-and-mouth disease viruses isolated from Afghanistan in 2003–2005. Virus Genes. 2008;36:401–13. PubMed DOI: 10.1007/s11262-008-0206-4

3. Marquardt O, Rahman MM, Freiberg B. Genetic and antigenic variance of foot-and-mouth disease virus type Asia1. Arch Virol. 2000;145:149–57. PubMed DOI: 10.1007/s007050050011

4. Valarcher J-F, Leforban Y, Rweyemamu M, Roeder PL, Gerbier G, Mackay DKJ, et al. Incursions of foot-and-mouth disease virus into Europe between 1985 and 2006. Transbound Emerg Dis. 2008;55:14–34. PubMed

5. Reddy GR, Tratschin JD, Natarajan C, Suryanarayana VV. Capsid protein-encoding (P1) sequence of foot and mouth disease virus type Asia 1 ind 63/72. Virus Genes. 1999;18:161–8. PubMed DOI: 10.1023/A:1008076805196

6. Sanyal A, Mohapatra JK, Kumar RM, Biswas S, Hemadri D, Tosh C, et al. Complete nucleotide sequence analysis of a vaccine strain and a field isolate of foot-and-mouth disease virus serotype Asia1 with an insertion in VP1 genomic region. Acta Virol. 2004b;48:159–66. PubMed

7. Gurumurthy CB, Sanyal A, Venkataramanan R, Tosh C, George M, Hemadri D. Genetic diversity in the VP1 gene of foot-and-mouth disease virus serotype Asia 1. Arch Virol. 2002;147:85–102. PubMed DOI: 10.1007/s705-002-8304-y

8. Sanyal A, Hemadri D, Tosh C, Bandyopadhyay SK. Emergence of a novel subgroup within the widely circulating lineage of foot-and-mouth disease virus serotype Asia 1 in India. Res Vet Sci. 2004;76:151–6. PubMed DOI: 10.1016/j.rvsc.2003.09.002

9. Carrillo C, Tulman ER, Delhon G, Lu Z, Carreno A, Vagnozzi A, et al. Comparative genomics of foot-and-mouth disease virus. J Virol. 2005;79:6487–504. PubMed DOI: 10.1128/JVI.79.10.6487-6504.2005

10. Khounsy S, Conlan JV, Gleeson LJ, Westbury HA, Colling A, Paton DJ, et al. Molecular epidemiology of foot foot-and and-mouth mouth disease viruses from South East Asia 1998-2006: The Lao perspective. Vet Microbiol. In press. PubMed

11. Knowles NJ, Davies PR, Henry T, O'Donnell V, Pacheco JM, Mason PW. Emergence in Asia of foot-and-mouth disease viruses with altered host range: characterization of alterations in the 3A protein. J Virol. 2001;75:1551–6. PubMed DOI: 10.1128/JVI.75.3.1551-1556.2001

12. Sosnovtsev SV, Onishchenko AM, Petrov NA, Mamaeva NV, Kalashnikova TI, Perevozchikova NA, et al. Primary structure of the gene for VP1 protein of the foot-and-mouth disease virus of Asia 1 serotype [in Russian]. Mol Gen Mikrobiol Virusol. 1989;12:44–6. PubMed
